# Estimates of Stillbirths, Neonatal Mortality, and Medically Vulnerable Live Births in Amhara, Ethiopia

**DOI:** 10.1001/jamanetworkopen.2022.18534

**Published:** 2022-06-24

**Authors:** Grace J. Chan, Frederick G. B. Goddard, Bezawit Mesfin Hunegnaw, Yahya Mohammed, Mesfin Hunegnaw, Sebastien Haneuse, Chalachew Bekele, Delayehu Bekele

**Affiliations:** 1 Division of Medicine Critical Care, Boston Children’s Hospital, Department of Pediatrics, Harvard Medical School, Boston, Massachusetts; 2 Department of Epidemiology, Harvard T.H. Chan School of Public Health, Boston, Massachusetts; 3 Department of Pediatrics and Child Health, St Paul’s Hospital Millennium Medical College, Addis Ababa, Ethiopia; 4 HaSET, St Paul’s Hospital Millennium Medical College, Addis Ababa Ethiopia; 5 Department of Biostatistics, Harvard T.H. Chan School of Public Health, Boston, Massachusetts; 6 Department of Obstetrics and Gynecology, St Paul’s Hospital Millennium Medical College, Addis Ababa, Ethiopia

## Abstract

**Importance:**

Data on birth outcomes and early mortality are scarce, especially in settings with limited resources. Total births, both stillbirths and live births, are often not counted, yet such data are critical to allocate resources and target interventions to improve survival.

**Objective:**

To estimate the prevalence of stillbirths, neonatal deaths, and medically vulnerable phenotypes, such as preterm births, small-for-gestational-age (SGA), large-for-gestational-age (LGA), and low-birth-weight (LBW) births, in a setting where these key indicators remain largely unknown.

**Design, Setting, and Participants:**

This prospective pregnancy cohort study of women and their newborns was conducted between December 12, 2018, and November 5, 2020. The study was conducted in North Shewa Zone, Amhara, Ethiopia. Data were analyzed from July 2021 to May 2022.

**Main Outcomes and Measures:**

Pregnancy status, gestational age, birth weight, and vital status were measured to estimate the prevalence of stillbirths, live births, and medically vulnerable live births (ie, preterm, SGA, LGA, and LBW births). For mortality outcomes, the prevalence of neonatal (overall, early, and late) and perinatal mortality were estimated.

**Results:**

Among the 2801 enrolled women, the median (IQR) age at conception was 26.5 (22.2-31.0) years, and the median (IQR) gestational age at enrollment was 24 (17-31) weeks. Of the 2628 women (93.8%) with outcome data, 101 pregnancies (3.8%) resulted in an early loss (<28 gestational weeks). Among the 2527 remaining pregnant women, there were 2518 births between 28 and less than 46 weeks’ gestation; 2459 (97.7%; 95% CI, 97.0%-98.2%) were live births and 59 (2.3%; 95% CI, 1.8%-3.0%) were stillbirths. Many newborns (41.7%) were born preterm, SGA, LGA, or LBW. The estimated prevalence was 15.1% (95% CI, 13.7%-16.6%) for preterm births, 23.1% (95% CI, 21.3%-25.1%) for SGA births, 10.6% (95% CI, 9.3%-12.1%) for LGA births, and 9.4% (95% CI, 8.2%-10.8%) for LBW births. Among live births, the overall prevalence of neonatal mortality was 3.1% (95% CI, 2.5%-3.9%); mortality was higher among preterm births (7.2%; 95% CI, 4.9%-10.4%), LBW births (12.2%; 95% CI, 8.2%-17.7%), and SGA births (4.1%; 95% CI, 2.6%-6.5%). The prevalence of early neonatal mortality was almost twice as high as the prevalence of late neonatal mortality. The perinatal mortality prevalence was 4.3% (95% CI, 3.6%-5.2%), with a 1.2:1 ratio of stillbirths to first-week deaths.

**Conclusions and Relevance:**

These findings have important implications for newborn health and survival. For policy makers and programmers, accurate data on key indicators of neonatal health provide information for resource allocation and to evaluate progress. For researchers, the findings underlie the importance for further research to develop and deliver interventions that improve health outcomes.

## Key Points

**Question:**

What is the prevalence of stillbirths, neonatal deaths, and medically vulnerable newborns (ie, those who are born preterm, small for gestational age, large for gestational age, or low birth weight) in Amhara, Ethiopia?

**Findings:**

In this cohort study of 2628 women and their newborns, with consistent recording of pregnancies and pregnancy outcomes, there was a high prevalence of stillbirths (2.3%) and neonatal mortality (3.1%). Many newborns (41.7%) were born preterm, small for gestational age, large for gestational age, or low birth weight.

**Meaning:**

For policy makers, programmers, and researchers, these findings have implications for resource allocation, evaluation of progress, and development and delivery of interventions to improve health outcomes.

## Teaser

This cohort study estimates the prevalence of stillbirths and neonatal deaths and infants born preterm, small for gestational age, large for gestational age, and low birth weight in Amhara, Ethiopia.

## Introduction

Globally, each year an estimated 2.0 million stillbirths occur^1^ and 2.4 million newborns die during the neonatal period, the first 28 days of life, contributing to 46% of deaths among children younger than 5 years.^2^ Fifteen million newborns are born preterm before 37 weeks’ gestation,^3^ 20 million are low birth weight (LBW; <2500 g), and 23 million newborns are born small for gestational age (SGA).^4^ Together, stillbirths, neonatal mortality, and medically vulnerable newborns born preterm, LBW, and SGA account for substantial long-term loss of human potential. Small size at birth, due to preterm birth and/or SGA, is the risk factor associated with more than 80% of neonatal deaths and increases the risk of postneonatal mortality, growth failure, neurodevelopmental impairment, and noncommunicable diseases.^5^ The risk of neonatal mortality is up to 10 times higher among preterm vs term newborns, and complications from preterm birth account for the greatest number of deaths among children younger than 5 years globally.^6,7^ Among newborns who are SGA, the risk of neonatal mortality is twice as high, and the mortality risk of newborns who are both preterm and SGA is 15 times higher than that of newborns with either characteristic alone.^8^ Neonates who are large for gestational age (LGA) have a higher risk of metabolic disease later in life.^9,10^

Improvements in health are based on identifying the most medically vulnerable individuals, preventing development of disease, delivering interventions, allocating appropriate resources, and monitoring progress. However, estimates of stillbirths, mortality, and vulnerable phenotypes are limited because of gaps in available data sources and quality.^11^ Registration of births and deaths is affected by perceptions of viability and social and economic factors, leading to imperfect denominators and misclassification of stillbirths vs live births.^6^ Fewer than 3% of births are formally registered with civil authorities in Ethiopia.^12^ The definitions and gestational age cutoffs for stillbirths vary by country and context, with some registries using 20 weeks or more, 28 weeks or more, or 1000 g or more. Consistent recording of all pregnancy outcomes, including stillbirths, and standardized definitions are critical to advance the understanding and the monitoring of trends in all settings.^6^ To improve the reporting of births and deaths, we conducted a pregnancy and birth cohort with pregnancy surveillance every 3 months and close follow-up after delivery with vital status data.^13^ Our study aims to estimate the number and prevalence of stillbirths, neonatal deaths, and medically vulnerable newborns (ie, who are born preterm, LBW, SGA, and LGA) in Ethiopia.

## Methods

### Study Population

This study was conducted at the Birhan field site, which was established in 2018 for community-based and facility-based research and research training with a specific focus on maternal and child health. The catchment area covers 16 kebeles (ie, lowest administrative unit) in 2 districts in North Shewa Zone, 130 km north of Addis Ababa, Ethiopia. The area is rural and semiurban, with highland and lowland topography and varying climates. Each household and household member have unique identification numbers to allow for longitudinal follow-up. Birhan includes approximately 19 000 households and a population of 78 000.^14^ Nested within the field site is an open dynamic pregnancy and birth cohort.^15^ The sample population for this study included women enrolled during pregnancy between December 12, 2018, and March 31, 2020, and their newborns. The last recorded delivery was on November 5, 2020. To estimate birth and mortality outcomes, we included all women in their first pregnancy during this period who delivered after 28 weeks’ gestation and their newborns born into the cohort. The 28-week gestation cutoff was used because in Ethiopia stillbirths are considered at 28 weeks or more. The study received ethical approval from St Paul’s Hospital Millennium Medical College and Harvard School of Public Health institutional review boards. All participants provided written informed consent. We followed the Strengthening the Reporting of Observational Studies in Epidemiology (STROBE) reporting guidelines.^16^

### Data Collection

Within the context of Birhan field site activities, house-to-house surveillance was conducted every 3 months to collect data on individual demographic and health information, including pregnancy status. Eligible pregnant women who consented were enrolled into the cohort. Women were subsequently followed up with scheduled home visits and facility visits when they presented for antenatal care visits or delivery. Home visits were conducted every 3 months until 32 weeks’ gestation, and then every 2 weeks. After 36 weeks, women were followed up every week through delivery. Gestational age was estimated by ultrasonography, maternal recall of last menstrual period (LMP), fundal height measurement, or maternal report of pregnancy in months. Vital status was recorded at each visit. In health facilities, Apgar scoring by health workers was used to assess the condition of newborn. In the community, study data collectors assessed signs of life at birth (breathing or cry or movement at birth) through maternal report. Newborn weight was measured with a Seca 354 digital scale with accuracy to the nearest 1 g and using the tare function to adjust for any additional weight. Enrollment and in-person data collection were conducted from December 2018 until end of March 2020 when field data collection activities were affected by COVID-19. During the COVID-19 phase, the data collection method was modified to telephone-based follow-up for pregnant women who were already enrolled to the cohort to monitor pregnancy status, illnesses, and pregnancy or birth outcomes through November 5, 2020.^15^

### Outcomes

We defined stillbirth as a birth with no signs of life (eg, no heartbeat, or Apgar score of 0) at or after 28 weeks of pregnancy.^17^ Among live births, we examined 3 vulnerable outcomes (preterm, LBW, and SGA) according to gestational age and/or birth weight. Live births at 46 weeks’ gestation or more were excluded as implausible gestational ages at birth. Preterm births were defined as live births before 37 weeks of gestation or fewer than 259 days from the first date of a woman’s LMP.^18^ LBW was defined as live births born less than 2500 g. To estimate the prevalence of LBW births, newborns with birth weight measurements above the 99th percentile were excluded. SGA was defined as live births below the 10th percentile of weight for gestational age using Intergrowth-21 standards.^19,20^ To estimate the prevalence of SGA, newborns with available birth weight (≤99th percentile) and gestational ages between 28 to less than 43 weeks’ gestation (the range for the Intergrowth-21 standards) were included. We defined LGA as live births above the 90th percentile of weight for gestational age using Intergrowth-21 standards.^19,20^

Neonatal mortality was defined as a death among live births before reaching 28 days of life, with early neonatal mortality defined as deaths on days 0 to 6 and late neonatal mortality as deaths on days 7 to 27 of life. Perinatal mortality was defined as stillbirths plus early neonatal mortality. Newborns who were lost to follow-up were excluded from primary analyses (see sensitivity analyses in the Results section). We defined newborns as having been lost to follow-up for neonatal mortality if there was no indication of death between days 0 and 27 and there was no vital status information suggesting they were alive after day 27. For early and perinatal mortality, newborns were considered lost to follow-up if there was no indication of death between days 0 and 6 and there was no vital status information suggesting they were alive after day 6.

### Statistical Analysis

We described population characteristics for women included in the study and estimated the unadjusted, crude prevalence of stillbirths and live births. Among live births, we estimated preterm, SGA, LGA, and LBW births. For mortality outcomes, we estimated the prevalence of neonatal (overall, early, and late) and perinatal mortality. Estimates of neonatal mortality outcomes were stratified by birth outcome status, specifically preterm, SGA, and LBW. Throughout we constructed 95% CIs using the Agresti and Coull method as described by Brown et al.^21^ We conducted our primary analysis among all births. Because multiple births (eg, twins) have a higher risk of morbidity and mortality, we repeated the descriptive analysis for singleton births.

Gestational age at birth was estimated using the best available method from ultrasonography measurements, maternal report of LMP, maternal report in months, and fundal height. Gestational age was estimated using a gestational age hierarchy, adapted from the American College of Obstetricians and Gynecologists guidelines^22^ and Global Library of Women’s Medicine^23^ (eAppendix 1 in the Supplement).

Missing data were encountered because of loss to follow-up, missing measurements, and excluded measurements that were outside a defined range of reasonable values. To investigate this, we performed sensitivity analyses to estimate a possible prevalence range for each outcome using different scenarios of outcome prevalence in the missing data. We first estimated outcome cases in the missing data by considering changes in outcome prevalence from half (−50%) to triple (200%) the prevalence we found in the nonmissing data. We then combined these modeled cases with the cases in the nonmissing data and re-estimated the overall prevalence and 95% CI. For all outcomes, except for stillbirth, we conservatively assumed that all pregnancies lost to follow-up could have resulted in live births, hence resulting in a potentially missed preterm, SGA, or LBW birth outcome or a missed mortality outcome. All missing and excluded measurements were also included in the sensitivity analyses as a potential case for each outcome. Data were analyzed from July 2021 to May 2022. All analyses were in R statistical software version 4.0.5 (R Project for Statistical Computing).^24^

## Results

This study enrolled 2801 women with a median (IQR) age at conception of 26.5 (22.2-31.0) years and median (IQR) gestational age at enrollment of 24 (17-31) weeks. Women had a median (IQR) of 2 (0-3) prior pregnancies and a median (IQR) of 3.2 (1.8-5.3) years between the current and last pregnancy. Most women resided in rural areas (2250 of 2801 women [80.3%]) and gave birth in a facility (1854 of 2527 women [73.4%]). Approximately one-half of the women (1478 of 2795 women [52.9%]) received formal education, and approximately one-third (895 of 2801 women [32.0%]) were primiparous (Table 1).

**Table 1.** Pregnancy Cohort Summary Characteristics

Characteristic	Patients, No./total No. (%) (N = 2801)
Completed follow-up	2628/2801 (93.8)
Residing in rural areas	2250/2801 (80.3)
Formal education	1478/2795 (52.9)
Facility delivery	1854/2527 (73.4)
Primiparous	895/2801 (32.0)
Maternal age at conception, median (IQR), y (n = 2794)	26.5 (22.2-31.0)
Gestational age at enrollment, median (IQR), wk (n = 2799)	23.9 (17.3-31.1)
Prior pregnancies, median (IQR), No. (n = 2801)	2.0 (0.0-3.0)
Time between current and last pregnancy, median (IQR), y (n = 1468)	3.2 (1.8-5.3)

**Figure 1.**
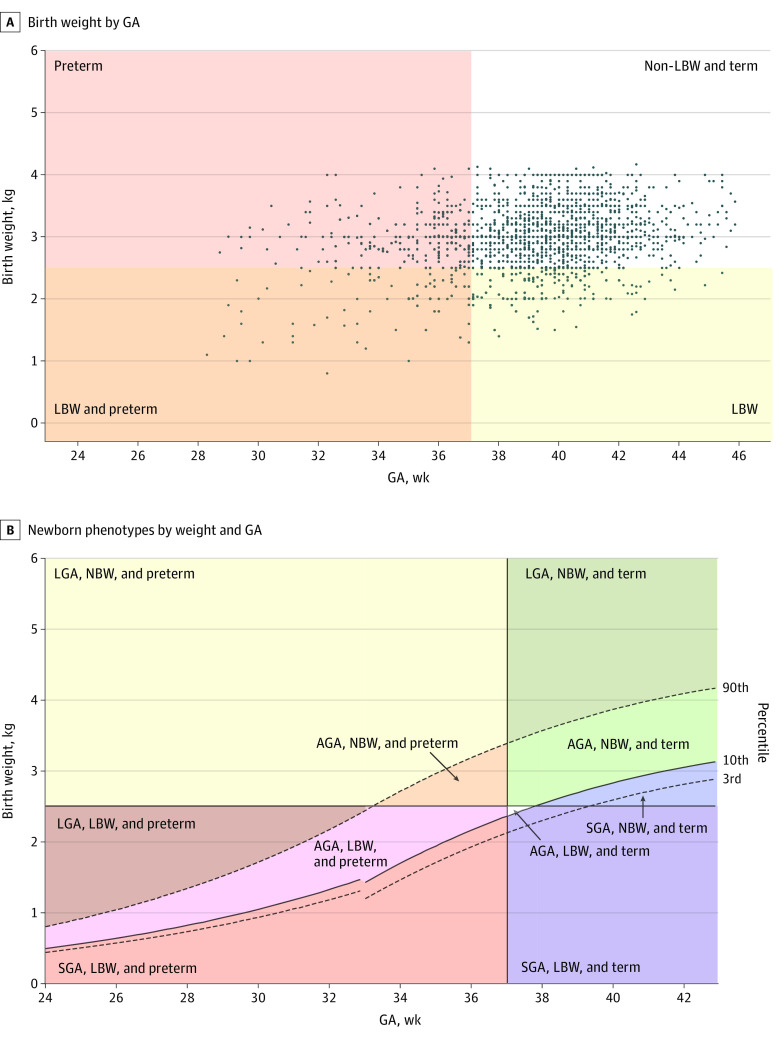
Birth Weight by Gestational Age (GA) and Newborn Phenotypes by Weight and GA Data are shown for 1904 newborns. One hundred seventeen newborns (6.1%) were born large for gestational age (LGA), normal birth weight (NBW), and preterm; 110 (5.8%) were born appropriate for gestational age (AGA), NBW, and preterm; 6 (0.3%) were born LGA, low birth weight (LBW), and preterm; 39 (2.0%) were born AGA, LBW, and preterm; 14 (0.7%) were born small for gestational age (SGA), LBW, and preterm; 79 (4.1%) were born LGA, NBW, and term; 1110 (58.3%) were born AGA, NBW, and term; 303 (15.9%) were born SGA, NBW, and term; 3 (0.2%) were born AGA, LBW, and term; and 123 (6.5%) were born SGA, LBW, and term.

Among the 2628 pregnancies (93.8%) with complete follow-up, 101 (3.8%) resulted in recorded miscarriages or abortions (pregnancy loss at <28 weeks). The remaining 2527 pregnancies resulted in 2564 births; 46 births were less than 28 weeks’ gestation or 46 or more weeks’ gestation and were excluded from the analysis. Among the 2518 remaining births, 2459 (97.7%; 95% CI, 97.0%-98.2%) were live births and 59 (2.3%; 95% CI, 1.8%-3.0%) were stillbirths (Table 2). Among the live births, there was a wide distribution of gestational ages. At any given gestational age, there was also wide distribution of birth weights. Figure 1A plots the distribution of birth weights by gestational age. There was also missingness in our data, with 477 live births (18.9%) missing birth weight measurements. Missing data details can be found in eAppendix 2 in the Supplement. Among 2012 births with birth weight data, most newborns were weighed within 3 days of birth, with 128 weights (5.1%) measured between 4 and 7 days after birth.

**Table 2.** Birth Outcomes Among All Births

Outcome	Births, No./total No. (%) [95% CI] (N = 2518)
Live birth	2459/2518 (97.7) [97.0-98.2]
Preterm birth	371/2459 (15.1) [13.7-16.6]
Low birth weight	190/2012 (9.4) [8.2-10.8]
Small for gestational age	440/1904 (23.1) [21.3-25.1]
Large for gestational age	202/1904 (10.6) [9.3-12.1]
Stillbirth	59/2518 (2.3) [1.8-3.0]

The estimated prevalence of neonates born preterm, SGA, LGA, or LBW was high (794 neonates; 41.7%; 95% CI, 39.5%-43.9%). Only 1110 of 1904 neonates (58.3%; 95% CI, 56.1%-60.5%) were appropriate for gestational age (AGA; ie, above the 10th and less than the 90th percentile), term (≥37 weeks’ gestational age), and normal birth weight (≥2500 g). An estimated 371 newborns (15.1%; 95% CI, 13.7%-16.6%) were preterm, 440 (23.1%; 95% CI, 21.3%-25.1%) were SGA, and 190 (9.4%; 95% CI, 8.2%-10.8%) were LBW. Among preterm births, 266 of 371 (71.7%; 95% CI, 66.9%-76.0%) were late preterm (34 to <37 weeks). Preterm classifications (very, moderate, and late) are described in eTable 1 in the Supplement. There were 202 newborns (10.6%; 95% CI, 9.3%-12.1%) who were LGA (>90th percentile). Nine percent of newborns were born with more than 1 vulnerable outcome: 14 (0.7%; 95% CI, 0.4%-1.2%) were born preterm, LBW, and SGA; 45 (2.4%; 95% CI, 1.8%-3.2%) were born preterm and LBW; and 123 (6.5%; 95% CI, 5.4%-7.7%) were born SGA and LBW. Figure 1B illustrates the different newborn phenotypes. Among the 72 twins, we found a higher proportion of adverse outcomes; 2 (2.8%) were stillbirths, and the prevalence of preterm was 34.3% (24 of 70 newborns), that of SGA was 49.2% (30 of 61 newborns), and that of LBW was 59.0% (36 of 61 newborns). Details on birth outcomes among singleton births can be found in eTable 2 in the Supplement.

Among all 2411 live births (singletons and twins) included in the analysis with completed 27-day neonatal follow-up, there were 75 (3.1%; 95% CI, 2.5%-3.9%) neonatal deaths (Table 3). The prevalence of neonatal mortality was 26 of 362 newborns (7.2%; 95% CI, 4.9%-10.4%) among preterm births, 23 of 188 newborns (12.2%; 95% CI, 8.2%-17.7%) among LBW births, and 18 of 434 newborns (4.1%; 95% CI, 2.6%-6.5%) among SGA births. Mortality among newborns who were AGA and term was low (16 of 1089 newborns; 1.5%; 95% CI, 0.9%-2.5%). The rate of mortality with 1 or more adverse outcome was 4.7% (37 of 780 newborns; 95% CI, 3.4%-6.5%). Mortality was higher among newborns with more than 1 outcome: there were 14 deaths among 59 newborns (23.7%; 95% CI, 14.6-36.1) who were born both preterm and LBW. The prevalence of early neonatal mortality at less than 7 days of life (48 of 2425 newborns; 2.0%; 95% CI, 1.5%-2.6%) was almost twice as high as the prevalence of late neonatal mortality (27 of 2411 newborns; 1.1%; 95% CI, 0.8%-1.6%). The perinatal mortality prevalence was 4.3% (107 of 2484 newborns; 95% CI, 3.6%-5.2%) when combining the neonatal mortality among 2484 live births with completed 6-day neonatal follow-up (48 deaths) and 59 stillbirths; there was a 1.2:1 ratio of stillbirths to first-week deaths. In total, there were 134 (5.4%; 95% CI, 4.6%-6.4%) stillbirths and neonatal deaths. Details on neonatal and perinatal mortality among singleton births can be found in eTable 3 in the Supplement. Among the 70 twin live births, we found a higher proportion of neonatal mortality (9 deaths; 12.9%; 95% CI, 6.7%-22.9%).

**Table 3.** Neonatal and Perinatal Mortality Among Preterm, Low-Birth-Weight, and Small-for-Gestational Age Newborns Among All Births

Type of birth	Births, No./total No. (%) [95% CI]		
	Neonatal mortality	Early neonatal mortality	Late neonatal mortality
All births	75/2411 (3.1) [2.5-3.9]	48/2425 (2.0) [1.5-2.6]	27/2411 (1.1) [0.8-1.6]
Preterm births			
Total	26/362 (7.2) [4.9-10.4]	14/366 (3.8) [2.2-6.4]	12/362 (3.3) [1.8-5.8]
Late preterm (34 to <37 wk)	7/259 (2.7) [1.2-5.6]	4/263 (1.5) [0.5-4.0]	3/259 (1.2) [0.2-3.5]
Moderately preterm (32 to <34 wk)	7/60 (11.7) [5.5-22.5]	3/60 (5.0) [1.2-14.3]	4/60 (6.7) [2.2-16.4]
Very preterm (<32 wk)	12/43 (27.9) [16.6-42.8]	7/43 (16.3) [7.8-30.3]	5/43 (11.6) [4.6-24.9]
Low-birth-weight births	23/188 (12.2) [8.2-17.7]	13/188 (6.9) [4.0-11.6]	10/188 (5.3) [2.8-9.6]
Small-for-gestational-age births	18/434 (4.1) [2.6-6.5]	11/434 (2.5) [1.4-4.5]	7/434 (1.6) [0.7-3.4]
Perinatal mortality	107/2484 (4.3) [3.6-5.2]	NA	NA
Neonatal mortality and stillbirths	134/2470 (5.4) [4.6-6.4]	NA	NA

Abbreviation: NA, not applicable.

**Figure 2.**
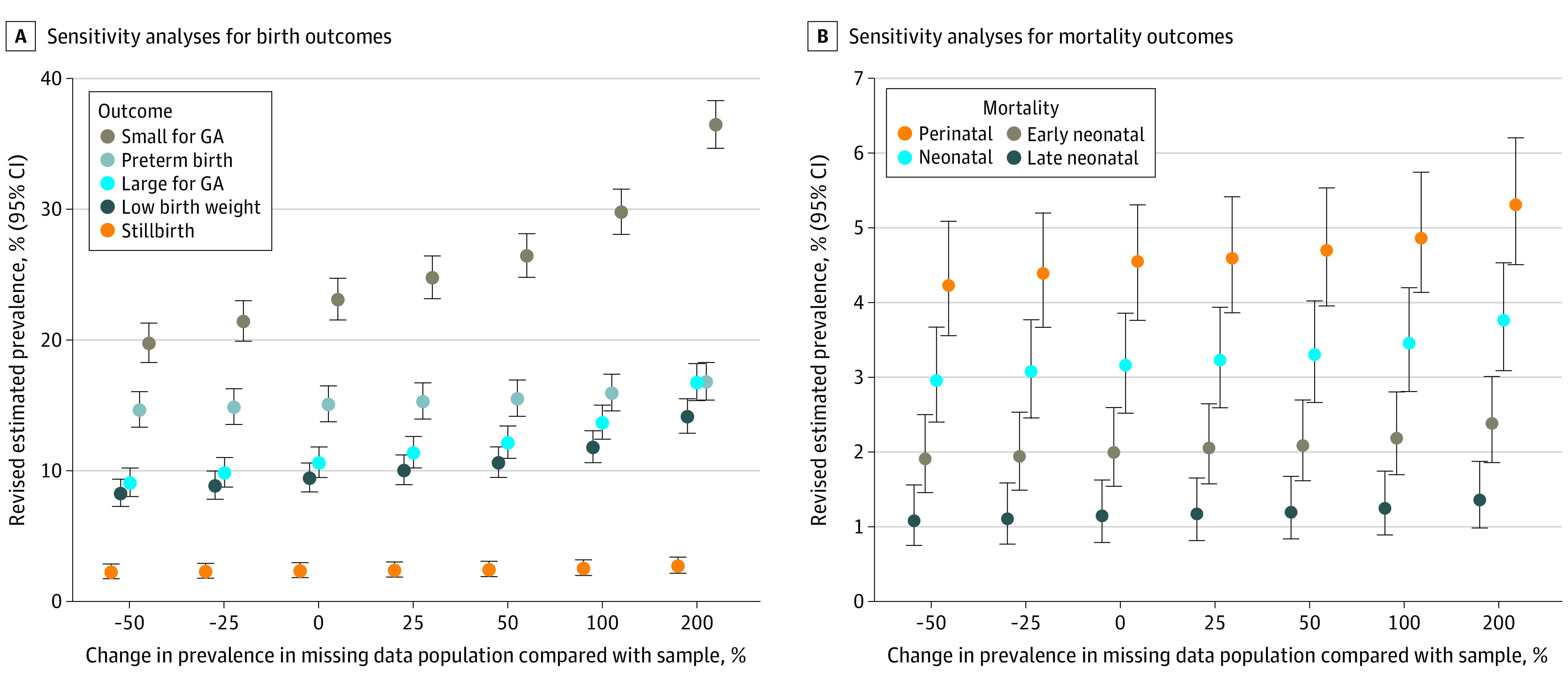
Sensitivity Analysis for Birth and Mortality Outcomes For each change in mortality prevalence in the population with missing data vs the sample, the revised estimated prevalence of mortality was 1.1%, 1.1%, 1.1%, 1.1%, 1.2%, 1.2%, and 1.3% for late neonatal mortality; 1.9%, 1.9%, 2.0%, 2.0%, 2.1%, 2.2%, and 2.4% for early neonatal mortality; 3.0%, 3.0%, 3.1%, 3.2%, 3.3%, 3.4%, and 3.7% for neonatal mortality; and 4.3%, 4.4%, 4.5%, 4.6%, 4.7%, 4.9%, and 5.3% for perinatal mortality. GA indicates gestational age.

Sensitivity analyses with data from all births suggested that if the prevalence of stillbirths in pregnancies lost to follow-up ranged from half to triple the prevalence found in the population with completed follow-up (ie, 1.1% to 6.6%), then the point estimates for prevalence of stillbirth ranged from 2.2% to 2.7% (Figure 2A). For the other birth outcomes, where missing data are not limited to incomplete follow-up but also include missing and out-of-range measurements, the point estimates from the sensitivity analyses for prevalence ranged from 14.7% to 16.8% for preterm births, 8.3% to 14.2% for LBW births, and 19.8% to 36.5% for SGA births.

Sensitivity analyses indicated that if the prevalence of neonatal mortality in pregnancies lost to follow-up ranged from half to triple the prevalence found in the population with completed follow-up (ie, 1.5% to 9.3%), then the point estimates for prevalence of neonatal mortality ranged from 3.0% to 3.7% (Figure 2B). For the other mortality outcomes, the point estimates from the sensitivity analyses for prevalence ranged from 1.9% to 2.4% for early neonatal mortality, 1.1% to 1.3% for late neonatal mortality, and 4.3% to 5.3% for perinatal mortality.

## Discussion

The findings of this cohort study provide estimates of the prevalence of stillbirths, neonatal mortality, and medically vulnerable phenotypes in Amhara, Ethiopia. We demonstrate the capacity to generate evidence for these key indicators using high-quality measurements (eg, nearly complete denominators and ultrasonography gestational age dating) in settings where these indicators have been largely unknown or modeled and there are major data gaps. The estimated prevalence of stillbirths and neonatal mortality in our study was similar to studies in South Asia but higher than other studies in sub-Saharan Africa,^11^ suggesting the importance of context-specific findings and the need for additional data to contribute to global and regional estimates. Neonatal mortality was higher among medically vulnerable newborns and highest among preterm and LBW newborns, compared with preterm only, LBW, or AGA and term newborns. Similar to other studies, the burden of neonatal mortality was higher during the first 7 days of life, the early neonatal period.^11^ We found a large proportion of SGA newborns, suggesting that newborns in this population are smaller than international standards. We found an expected proportion of LGA newborns and propose global estimates to include LGA phenotypes in addition to SGA in assessing risk of health conditions into childhood and adulthood. In our study population, there was a wide distribution of gestational ages, and, at any given gestational age, there was wide variation of newborn weights, reflecting an expected distribution of these metrics in a setting where more than 95% of deliveries are spontaneous.

Our results have important implications for newborn health and survival. High perinatal mortality highlights the need for further research to detect and manage women at risk to prevent adverse outcomes. We can improve identification of vulnerable newborns (preterm, SGA, LGA, and LBW newborns) who have a high risk of dying or later health conditions by investing in better gestational age and birth weight measurements and registering all births. Interventions that improve care and survival of these newborns should be prioritized. Early initiation of breastfeeding, kangaroo mother care,^25^ treatment of neonatal sepsis,^26^ neonatal resuscitation,^27^ and bubble continuous positive airway pressure are cost-effective interventions that improve survival,^28^ yet these interventions remain largely inaccessible to newborns in settings that carry the highest mortality burden. For policy makers and programmers, our results highlight the compelling need for investments in maternal, fetal, and newborn health. To evaluate progress, we can assess temporal changes in the distribution of stillbirths, preterm, LBW, SGA, and neonatal mortality and assess the impact of programmatic interventions and events such as the COVID-19 pandemic. For researchers, our findings underlie the importance for further research to develop interventions for prevention of preterm birth and fetal growth restriction. Preterm birth is associated with maternal risk factors, including infection and undernutrition, but interventions treating maternal infections and nutritional supplementation have limited evidence to scale and varying effects across populations.^29^ Despite known associations between risk factors and preterm births, the biological basis for the timing of parturition is largely unknown^30,31,32^ and remains an urgent priority for research to better inform interventions.^33^ Future work should include accounting for early pregnancy losses due to miscarriages and inferential studies to investigate risk associations with adverse birth outcomes and risk prediction models.

### Strengths and Limitations

Our study contributes longitudinal data with pregnancy surveillance and follow-up to consistently record pregnancies and pregnancy outcomes with high-quality measures of birth weight and gestational age. Global estimates of stillbirths, neonatal mortality, and vulnerable phenotypes rely on models that use data from diverse sources such as cross-sectional surveys, health management information systems, and national census that have variable quality and methodological limitations.^11^ In Ethiopia, neonates are often not weighed after birth; 14% of births have a reported birth weight in Ethiopia.^12^ Although we captured birth weight data from more than 80% of births, many of which were missed because of challenges to in-person follow-up during COVID-19, limitations of this study include that a large proportion of birth weights were not recorded. Accurate gestational age measures are often missing without criterion standard assessments of early ultrasonography with fetal measurements in the first trimester. Gestational age measurements by maternal recall of the LMP remains the only available method in many settings, yet LMP has low accuracy due to variations in the lengths of individual menstrual cycles and biases in recall. Live preterm births may be counted as abortion or stillbirth without accurate gestational age dating, varying gestational age cutoff points for preterm definitions, or perceived nonviability leading to misclassification of outcomes.^6^

A strength of this study is that we present a nearly complete data set, with approximately 93.8% of pregnancies with completed follow-up. We weighed neonates born in the community and facility as soon as possible with digital scales. We introduced ultrasonography gestational age dating at the health facilities and reached approximately 20% of pregnancies before 16 weeks’ gestation. We adapted a best obstetric estimate combining ultrasonography and LMP to estimate gestational age. Sensitivity analyses demonstrated limited variation in mortality and stillbirth estimates based on different scenarios of outcome prevalence among missing data. We present our work as a potential model to scale nationally or in other low-income and middle-income country settings to generate estimates of these key health indicators.

## Conclusions

Our study contributes to the global estimates of pregnancy and birth outcomes and the substantial burden of stillbirths, mortality, and vulnerable phenotypes (ie, preterm, LBW, SGA, and LGA births). We look to forge close collaborations between researchers and policy makers and programmers to use data for impact, particularly to discover and deliver interventions that improve survival.

## Notes

Supplement. **eAppendix 1.** Gestational Age Hierarchy **eAppendix 2. **Missing Data Information **eTable 1.** Estimates of Preterm Classifications: Late Preterm (34 to <37 Weeks), Moderately Preterm (32 to <34 Weeks), and Very Preterm (<32 Weeks) **eTable 2.** Birth Outcomes Among Singleton Births **eTable 3.** Neonatal and Perinatal Mortality Among Preterm, Low Birth Weight and Small-for-Gestational Age Newborns Among Singleton Births
eReferences
