# Supplementary material for: Estimates of Stillbirths, Neonatal Mortality, and Medically Vulnerable Live Births in Amhara, Ethiopia
Source: JAMA Netw Open. 2022 Jun 24;5(6):e2218534. doi: 10.1001/jamanetworkopen.2022.18534 (PMC9233235; doi:10.1001/jamanetworkopen.2022.18534)
Supplement: Supplement. — eAppendix 1. Gestational Age Hierarchy eAppendix 2. Missing Data Information eTable 1. Estimates of Preterm Classifications: Late Preterm (34 to <37 Weeks), Moderately Preterm (32 to <34 Weeks), and Very Preterm (<32 Weeks) eTable 2. Birth Outcomes Among Singleton Births eTable 3. Neonatal and Perinatal Mortality Among Preterm, Low Birth Weight and Small-for-Gestational Age Newborns Among Singleton Births eReferences [file jamanetwopen-e2218534-s001.pdf]

Supplemental Online Content

Chan GJ, Goddard FGB, Hunegnaw BM, et al. Estimates of stillbirths, neonatal mortality, and medically vulnerable live births in Amhara, Ethiopia. *JAMA Netw Open*. 2022;5(6):e2218534. doi:10.1001/jamanetworkopen.2022.18534

eAppendix 1. Gestational Age Hierarchy
eAppendix 2. Missing Data Information
eTable 1. Estimates of Preterm Classifications: Late Preterm (34 to <37 Weeks), Moderately Preterm (32 to <34 Weeks), and Very Preterm (<32 Weeks)
eTable 2. Birth Outcomes Among Singleton Births
eTable 3. Neonatal and Perinatal Mortality Among Preterm, Low Birth Weight and Small-for-Gestational Age Newborns Among Singleton Births
eReferences

This supplemental material has been provided by the authors to give readers additional information about their work.

© 2022 Chan GJ et al. *JAMA Network Open.*

eAppendix 1. Gestational Age Hierarchy

The gestational age hierarchy is described below. Briefly, if an ultrasound (US) measurement before 16 weeks gestation is available, then that measurement is used to estimate gestational age. If US is only available after 16 weeks gestation, then self-reported last menstrual period (LMP) is used to as long as there is only a limited discrepancy between the LMP and US estimates. If no US estimates are available to estimate gestational age, then LMP is used, and if neither of those two methods are available, then fundal height is used over a maternal estimate (reported in months).

	**≤ 8 6/7 weeks**	**9 0/7 to 15 6/7 weeks**	**16 0/7 to 21 6/7 weeks**	**22 0/7 to 27 6/7 weeks**	**≥ 28 0/7 weeks**
**If US is available and LMP is known and reliable**	US GA at birth and LMP and US discrepancy ≤ 5 days and: Use US	LMP and US discrepancy ≤ 7 days: Use US	LMP and US discrepancy ≤ 10 days: Use LMP	LMP and US discrepancy ≤ 14 days: Use LMP	LMP and US discrepancy ≤ 21 days: Use LMP
	LMP and US discrepancy >5 days: Use US	LMP and US discrepancy >7 days: Use US	LMP and US discrepancy >10 days: Use US	LMP and US discrepancy >14 days: Use US	LMP and US discrepancy >21 days: Use US
**If LMP is unknown or unreliable**	Use US input				
**If US is unavailable**	Use LMP input				
**If US is unavailable and LMP is unknown or unreliable; Fundal height is known and reliable**	Fundal height measurement available at date of collection → Use fundal height*<br>*Preference earliest date of collection if multiple				
**Fundal height is unknown or unreliable**	Fundal height measurement is not available → Use maternal recall of GA in months				

eAppendix 2. Missing Data Information

The sample for SGA and LBW births do not match the total number of livebirths. Among 2,459 livebirths, there were 477 missing birth weights, 261 from the pause in in-person data collection due to COVID-19. The remaining 216 missing birth weights were due to data collectors not capturing births within seven days for weight measurements or because they were excluded due to being above the 99th percentile of birth weight measurements. There were 555 livebirths missing S/LGA measures with the majority due to missing birth weight data, and the remaining 108 having gestational ages between 43 and <46 weeks gestation, outside of the Intergrowth-21 standards that go up to <43 weeks gestation. {Villar, 2014 #16808;Villar, 2014 #16309}

© 2022 Chan GJ et al. *JAMA Network Open.*

eTable 1. Estimates of Preterm Classifications: Late Preterm 34 to <37 Weeks, Moderately Preterm (32 to <34 Weeks), and Very Preterm (<32 Weeks)

	N	n	%	95% CI
Preterm births	2459	371	15.1	13.7-16.6
Late preterm (34 to <37 weeks)	371	266	71.7	66.9-76.0
Moderately preterm (32 to <34 weeks)	371	62	16.7	13.2-20.9
Very preterm (<32 week)	371	43	11.6	8.7-15.3

eTable 2. Birth Outcomes Among Singleton Births

	N	n	%	95% CI
Singleton births	2446			
Livebirths	2446	2389	97.7	97.0 – 98.2
Preterm birth	2389	347	14.5	13.2 – 16.0
Low birth weight	1951	154	7.9	6.8 – 9.2
Small for gestational age	1843	410	22.2	20.4 – 24.2
Large for gestational age	1843	202	11.0	9.6 – 12.5
Still births	2446	57	2.3	1.8 – 3.0

eTable 3. Neonatal and Perinatal Mortality Among Preterm, Low Birth Weight and Small-for-Gestational Age Newborns Among Singleton Births

	Neonatal mortality				Early neonatal mortality				Late neonatal mortality			
	N	n	%	95% CI	N	n	%	95% CI	N	n	%	95% CI
Singleton live births	2341	66	2.8	2.2 – 3.6	2355	44	1.9	1.4 – 2.5	234 1	22	0.9	0.6 – 1.4
Preterm births	338	19	5.6	3.6 – 8.7	342	12	3.5	2.0 – 6.1	338	7	2.1	0.9 – 4.3
Late preterm (34 to <37 weeks)	243	6	2.5	1.0 – 5.4	247	4	1.6	0.5 – 4.2	243	2	0.8	0.0 – 3.1
Moderately preterm (32 to <34 weeks)	56	5	8.9	3.5 – 19.7	56	3	5.4	1.3 – 15.2	56	2	3.6	0.3- 12.8
Very preterm (<32 weeks)	39	8	20.5	10.5 – 35.8	39	5	12.8	5.1 – 27.2	39	3	7.7	1.9 – 21.0
Low birth weight births	152	17	11.2	7.0 – 17.3	152	11	7.2	4.0 – 12.6	152	6	3.9	1.6 – 8.5
Small for gestational age births	404	17	4.2	2.6 – 6.7	404	10	2.5	1.3 – 4.6	404	7	1.7	0.8 – 3.6
Perinatal mortality	2412	101	4.2	3.5 – 5.1								
Neonatal mortality and stillbirths	2398	123	5.1	4.3 – 6.1								

eReferences

1. Villar J, Papageorghiou AT, Pang R, et al. The likeness of fetal growth and newborn size across non-isolated populations in the INTERGROWTH-21st Project: the Fetal Growth Longitudinal Study and Newborn Cross-Sectional Study. *The lancet Diabetes & endocrinology*. 2014;2:781-92.
2. Villar J, Cheikh Ismail L, Victora CG, et al. International standards for newborn weight, length, and head circumference by gestational age and sex: the Newborn Cross-Sectional Study of the INTERGROWTH-21st Project. *Lancet*. Sep 6 2014;384(9946):857-68. doi:10.1016/S0140-6736(14)60932-6

© 2022 Chan GJ et al. *JAMA Network Open.*
